# Surgical Delay and Pathological Outcomes for Clinically Localized High-Risk Prostate Cancer

**DOI:** 10.1001/jamanetworkopen.2020.28320

**Published:** 2020-12-08

**Authors:** Leilei Xia, Ruchika Talwar, Raju R. Chelluri, Thomas J. Guzzo, Daniel J. Lee

**Affiliations:** 1 Division of Urology, Department of Surgery, University of Pennsylvania Perelman School of Medicine, Philadelphia; 2 Leonard Davis Institute of Health Economics, University of Pennsylvania, Philadelphia

## Abstract

**Importance:**

There is a lack of data evaluating the association of surgical delay time (SDT) with outcomes in patients with localized, high-risk prostate cancer.

**Objective:**

To investigate the association of SDT of radical prostatectomy and final pathological and survival outcomes.

**Design, Setting, and Participants:**

This cohort study used data from the US National Cancer Database (NCDB) and identified all patients with clinically localized (cT1-2cN0cM0) high-risk prostate adenocarcinoma diagnosed between 2006 and 2016 who underwent radical prostatectomy. Data analyses were performed from April 1 to April 12, 2020.

**Exposures:**

SDT was defined as the number of days between the initial cancer diagnosis and radical prostatectomy. SDT was categorized into 5 groups: 31 to 60, 61 to 90, 91 to 120, 121 to 150, and 151 to 180 days.

**Main Outcomes and Measures:**

The primary outcomes were predetermined as adverse pathological outcomes after radical prostatectomy, including pT3-T4 disease, pN-positive disease, and positive surgical margin. The adverse pathological score (APS) was defined as an accumulated score of the 3 outcomes (0-3). An APS of 2 or higher was considered a separate outcome to capture cases with more aggressive pathological features. The secondary outcome was overall survival.

**Results:**

Of the 32 184 patients included in the study, the median (interquartile range) age was 64 (59-68) years, and 25 548 (79.4%) were non-Hispanic White. Compared with an SDT of 31 to 60 days, longer SDTs were not associated with higher risks of having any adverse pathological outcomes (odds ratio [OR], 0.95; 95% CI, 0.80-1.12; *P* = .53), pT3-T4 disease (OR, 0.99; 95% CI, 0.83-1.17; *P* = .87), pN-positive disease (OR, 0.79; 95% CI, 0.59-1.06; *P* = .12), positive surgical margin (OR, 0.88; 95% CI, 0.74-1.05; *P* = .17), or APS greater than or equal to 2 (OR, 0.90; 95% CI, 0.74-1.05; *P* = .17). Longer SDT was also not associated with worse overall survival (for SDT of 151-180 days, hazard ratio, 1.12; 95% CI, 0.79-1.59, *P* = .53). Subgroup analyses performed for patients with very high-risk disease (primary Gleason score 5) and sensitivity analyses with SDT considered as a continuous variable yielded similar results.

**Conclusions and Relevance:**

In this cohort study of patients who underwent radical prostatectomy within 180 days of diagnosis for high-risk prostate cancer, radical prostatectomy for high-risk prostate cancer could be safely delayed up to 6 months after diagnosis.

## Key Points

**Question:**

Is delayed radical prostatectomy for high-risk prostate cancer associated with negative patient outcomes?

**Findings:**

In this cohort study of 32 184 patients from the US National Cancer Database who underwent radical prostatectomy within 180 days of diagnosis for high-risk prostate cancer, increased surgical delay time was not associated with higher risks of adverse pathological features (pT3-4 disease, node positivity, or positive margin).

**Meaning:**

These findings suggest that prostate cancer surgery can be safely delayed up to 6 months and should be considered as low priority compared with other emergent and cancer surgeries when health care resources need to be prioritized during special times, such as the coronavirus disease 2019 pandemic.

## Teaser

This cohort study examines the association of surgical delay time with pathological and survival outcomes among patients with localized, high-risk prostate cancer.

## Introduction

The coronavirus disease 2019 (COVID-19) pandemic has placed unprecedented strain on individual patients and the modern practice of health care globally.^1^ Cancer care has become more complex, as clinicians and patients must balance concerns regarding COVID-19 exposure and resource allocation against cancer progression and patient anxiety.^2,3^ Counseling surrounding the topic of surgical timing is of utmost importance. Although delays in surgical management for lower-risk prostate cancer will result in minimal harm to the patient, limited data exist on the oncological safety of delaying definitive treatment for high-risk and very-high-risk prostate cancer.^4^

On the basis of a single institutional study,^5^ the National Comprehensive Cancer Network (NCCN) released guidelines regarding the management of prostate cancer during the pandemic, stating that patients with unfavorable intermediate-risk to very-high-risk disease can safely delay further workup, staging, and definitive treatment up to 6 months. There are some other recommendations regarding delaying surgery for prostate cancer during COVID-19 pandemic published in the literature, but the evidence behind those recommendations was generally limited^2,6,7^

To address this need, we sought to analyze the safety of delays in surgical management, focusing on more aggressive, organ-confined disease. We queried a large, contemporary national database to determine the association of length of time from diagnosis to radical prostatectomy with pathological outcomes in patients with localized, high-risk prostate cancer. We hypothesized that delay in surgical management for these patients would not be associated with finding of more adverse pathological features on final histopathological analysis.

## Methods

### Data Source

We identified patients in the National Cancer Database (NCDB) with clinically localized (cT1-2cN0cM0) high-risk prostate adenocarcinoma diagnosed between 2006 and 2016 who underwent radical prostatectomy. The NCDB is a nationwide, hospital-based, comprehensive clinical data set that currently captures 70% of all newly diagnosed malignant entities in the US annually.^8^ Data from the NCDB are deidentified; thus, the study was exempted from institutional review board review by the University of Pennsylvania, and informed consent was waived in accordance with 45 CFR §46. The reporting of our methods was consistent with Strengthening the Reporting of Observational Studies in Epidemiology (STROBE) reporting guideline.

### Cohort Selection

**Figure 1.**
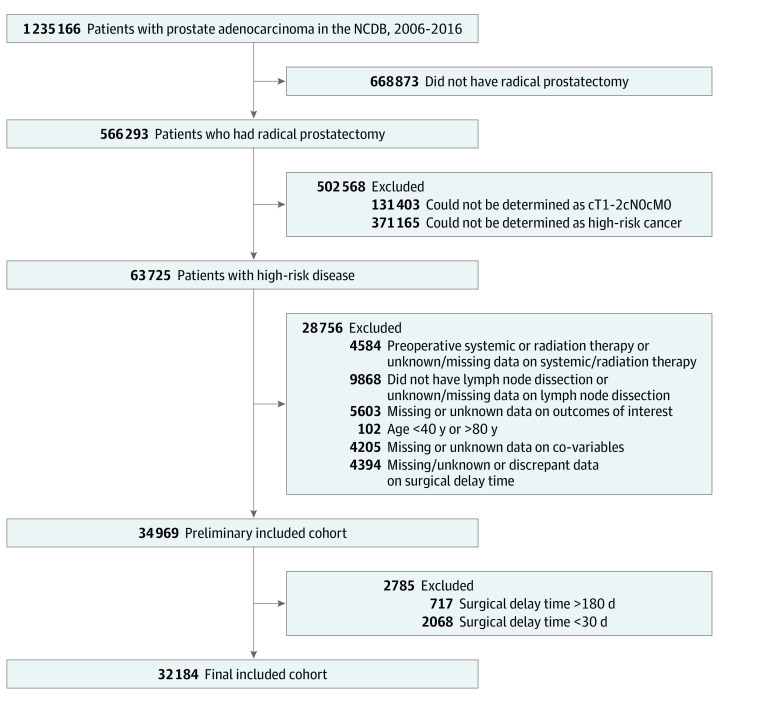
Flow Diagram of Cohort Selection NCDB indicates National Cancer Database.

Figure 1 shows the flow diagram of our final cohort selection. We only included clinically localized (cT1-2, cN0, cM0) disease because surgery (radical prostatectomy) is a well-established management option. Our inclusion criteria are consistent with the most recent American Urological Association, American Society for Radiation Oncology, Society of Urologic Oncology, and NCCN guidelines.^9,10,11^ Clinical stage T3 and T4 cancers are considered locally advanced disease and thus were excluded. Surgery as a management option for locally advanced cancer is not as clearly defined because of the increased risk of positive margins, lymph node metastases, and/or distant relapse.^12^ High-risk patients were defined as those with preoperative prostate-specific antigen (PSA) level greater than or equal to 20 ng/mL (to convert to micrograms per liter, multiply by 1) or biopsy grade group 4 to 5 (Gleason score 8-10).^9,10,11^ Patients who underwent preoperative systemic therapy or radiation therapy were excluded because those procedures are not standard of care, as were patients who did not have pelvic lymph node dissections at the time of radical prostatectomy. Cases with missing data in any of the outcomes of interest or covariables were also excluded. Patients younger than 40 years or older than 80 years were excluded because of rare occurrences and are usually outliers of clinical practice.

### Exposure of Interest

Surgical delay time (SDT) was defined as days between initial cancer diagnosis and radical prostatectomy. SDTs longer than 180 days were excluded because this was a very small cohort (2%) and could not be used for meaningful analyses if further stratified. SDTs 30 days or less were also excluded for 3 reasons. First, it represented a very small proportion of patients (6%), and most patients in our preliminary cohort had SDTs of 31 to 90 days (73.1%), which is consistent with common clinical practice. SDTs of 31 to 60 days can be used as a reasonable baseline reference. Urologists tend to recommend waiting at least 4 weeks after prostate needle biopsy before radical prostatectomy to allow biopsy-induced inflammation to subside.^13,14^ Second, stronger selection bias can be found in the early surgery groups, which were shown in NCDB studies on other cancers.^15,16^ Third, coding errors are more likely to be found with SDTs of 30 days or less because we even noticed some cases with SDTs of less than 15 days, which were unlikely to be correct considering the turnaround time of biopsy pathology results and surgery scheduling. SDT was grouped with 30-day (1 month) increments in accordance with previous studies and for the convenience of patient counseling.^16,17^

### Outcomes of Interest

The primary outcomes of interest of this study were predetermined as adverse pathological outcomes after radical prostatectomy, including pT3-T4 disease, pN-positive disease, and positive surgical margin (PSM). These are well-established cancer survival outcome surrogates. Any adverse pathological outcome was defined as having any of the 3 outcomes. We developed an adverse pathological score (APS) as an accumulated score of the 3 individual outcomes (0-3). We used an APS of 2 or higher as a separate outcome to capture more aggressive pathological features. The secondary outcome was overall survival (OS), which is the only available survival outcome in the NCDB.

### Statistical Analysis

Demographic, cancer, and facility characteristics were collected. Covariables included age, race/ethnicity, Charlson-Deyo comorbidity score, insurance, income level, education level, county type of patient’s residence, travel distance, clinical T stage, PSA level, Gleason score, facility type, facility location, and year of diagnosis. Travel distances greater than 250 miles were excluded to minimize the biases of patients who were too far away from residence when they sought care.^18^

Baseline characteristics were compared using χ^2^ and Kruskal-Wallis tests for categorical variables and continuous variables, respectively. Multivariable logistic regressions were used to assess the associations between SDT and primary outcomes. Kaplan-Meier curves were used to estimate the OS across different SDT groups, and multivariable Cox regressions were used to assess the associations between SDT and OS. Subgroup analyses were performed for patients with very-high-risk disease, which was defined as having primary Gleason score 5 on the basis of NCCN guidelines and data availability in the NCDB.^11^ Sensitivity analyses were performed for both the multivariable logistic regressions and Cox regressions, with SDT considered as a continuous variable (per 30-day increase). A 2-sided *P* < .05 was considered statistically significant. All analyses were performed using Stata statistical software version 15 (StataCorp). Data analyses were performed between April 1 and April 12, 2020.

## Results

### Study Cohorts

A total of 32 184 patients with high-risk prostate cancer were included in the final cohort. Baseline characteristics of the overall cohort stratified by SDT are presented in Table 1. Of the patients included in the study, the median (interquartile range) age was 64 (59-68) years, and 25 548 patients (79.4%) were non-Hispanic White. Most patients (25 383 patients [78.9%]) had a Charlson-Deyo comorbidity score of 0. In the overall cohort, 13 804 patients (42.9%) had an SDT of 31 of 60 days, 11 750 (36.5%) had an SDT of 61 to 90 days, 4489 (14.0%) had an SDT of 91 to 120 days, 1504 (4.7%) had an SDT of 121 to 150 days, and 637 (2%) had an SDT of 151 to180 days. A total of 2348 patients (7.3%) could be determined as having very-high-risk disease, including 330 (1.0%) with a Gleason score of 5 + 3, 1593 (5.0%) with a Gleason score of 5 + 4, and 425 (1.3%) with a Gleason score of 5 + 5.

**Table 1.** Baseline Characteristics of the Overall Included Cohort Stratified by SDT^a^

Characteristic	Patients, No. (%)					*P* value
	SDT 31-60 d (n = 13 804)	SDT 61-90 d (n = 11 750)	SDT 91-120 d (n = 4489)	SDT 121-150 d (n = 1504)	SDT 151-180 d (n = 637)	
Age, median (IQR), y	64 (59-68)	64 (59-68)	64 (59-68)	63 (58-68)	63 (58-67)	.01
Race/ethnicity						
Non-Hispanic White	11 577 (83.9)	9300 (79.1)	3347 (74.6)	923 (61.4)	401 (63.0)	<.001
Non-Hispanic Black	1357 (9.8)	1570 (13.4)	757 (16.9)	410 (27.3)	162 (25.4)	
Non-Hispanic other	431 (3.1)	374 (3.2)	152 (3.4)	72 (4.8)	24 (3.8)	
Hispanic	439 (3.2)	506 (4.3)	233 (5.2)	99 (6.6)	50 (7.8)	
Charlson-Deyo comorbidity score						
0	11 009 (79.8)	9271 (78.9)	3494 (77.8)	1133 (75.3)	476 (74.7)	<.001
1	2332 (16.9)	2014 (17.1)	817 (18.2)	282 (18.8)	125 (19.6)	
2	349 (2.5)	346 (2.9)	136 (3.0)	68 (4.5)	NR^b^	
≥3	114 (0.8)	119 (1.0)	42 (0.9)	21 (1.4)	NR^b^	
Insurance						
Private	7623 (55.2)	6183 (52.6)	2319 (51.7)	734 (48.8)	318 (49.9)	<.001
Medicare	5533 (40.1)	4841 (41.2)	1827 (40.7)	592 (39.4)	232 (36.4)	
Medicaid	282 (2.0)	326 (2.8)	146 (3.3)	77 (5.1)	45 (7.1)	
Other government	184 (1.3)	204 (1.7)	93 (2.1)	54 (3.6)	15 (2.4)	
Uninsured	182 (1.3)	196 (1.7)	104 (2.3)	47 (3.1)	27 (4.2)	
Education level %^c^						
≥21	1614 (11.7)	1518 (12.9)	680 (15.1)	271 (18.0)	127 (19.9)	<.001
13-20.9	2902 (21.0)	2701 (23.0)	1069 (23.8)	373 (24.8)	149 (23.4)	
7-12.9	4822 (34.9)	4043 (34.4)	1595 (35.5)	487 (32.4)	202 (31.7)	
<7	4466 (32.4)	3488 (29.7)	1145 (25.5)	373 (24.8)	159 (25.0)	
Income level, $^d^						
<38 000	1816 (13.2)	1577 (13.4)	722 (16.1)	279 (18.6)	122 (19.2)	<.001
38 000-47 999	2930 (21.2)	2533 (21.6)	963 (21.5)	309 (20.5)	129 (20.3)	
48 000-62 999	3798 (27.5)	3224 (27.4)	1209 (26.9)	395 (26.3)	154 (24.2)	
≥63 000	5260 (38.1)	4416 (37.6)	1595 (35.5)	521 (34.6)	232 (36.4)	
County type						
Metropolitan	11 339 (82.1)	9787 (83.3)	3797 (84.6)	1299 (86.4)	558 (87.6)	<.001
Urban	2144 (15.5)	1740 (14.8)	625 (13.9)	175 (11.6)	NR^b^	
Rural	321 (2.3)	223 (1.9)	67 (1.5)	30 (2.0)	NR^b^	
Travel distance, median (IQR), miles	13.7 (6-33.9)	14.7 (6.5-35.8)	14.6 (6.4-38.4)	13.9 (6.3-35.2)	14.3 (6.2-39.4)	<.001
Clinical T stage						
cT1	8977 (65.0)	7876 (67.0)	3066 (67.0)	1070 (68.3)	467 (73.3)	<.001
cT2	4827 (35.0)	3874 (33.0)	1423 (33.0)	434 (28.9)	170 (26.7)	
PSA level, ng/mL						
<10	8027 (58.1)	6686 (56.9)	2350 (56.9)	702 (46.7)	265 (41.6)	<.001
10 to <20	2241 (16.2)	1850 (15.7)	735 (16.4)	224 (14.9)	98 (15.4)	
≥20	3536 (25.6)	3214 (27.4)	1404 (31.3)	578 (38.4)	274 (43.0)	
Gleason score						
6	506 (3.7)	512 (4.4)	267 (5.9)	139 (9.2)	63 (9.9)	<.001
7	1737 (12.6)	1732 (14.7)	768 (17.1)	304 (20.2)	164 (25.7)	
8	6685 (48.4)	6148 (52.3)	2361 (52.6)	759 (50.5)	292 (45.8)	
9-10	4876 (35.3)	3358 (28.6)	1093 (24.3)	302 (20.1)	118 (18.5)	
Facility type						
Community	761 (5.5)	499 (4.2)	180 (4.0)	52 (3.5)	22 (3.5)	<.001
Comprehensive community	5532 (40.1)	4191 (35.7)	1385 (30.9)	412 (27.4)	163 (25.6)	
Academic or research	5756 (41.7)	5489 (46.7)	2318 (51.6)	824 (54.8)	386 (60.6)	
Integrated network	1755 (12.7)	1571 (13.4)	606 (13.5)	216 (14.4)	66 (10.4)	
Facility location						
New England	609 (4.4)	669 (5.7)	329 (7.3)	113 (7.5)	51 (8.0)	<.001
Middle Atlantic	1806 (13.1)	1827 (15.5)	777 (17.3)	262 (17.4)	100 (15.7)	
South Atlantic	2060 (14.9)	2120 (18.0)	860 (19.2)	311 (20.7)	132 (20.7)	
East North Central	3001 (21.7)	2262 (19.3)	820 (18.3)	226 (15.0)	105 (16.5)	
East South Central	1390 (10.1)	949 (8.1)	320 (7.1)	113 (7.5)	52 (8.2)	
West North Central	1709 (12.4)	1213 (10.3)	307 (6.8)	105 (7.0)	NR^b^	
West South Central	914 (6.6)	734 (6.2)	301 (6.7)	119 (7.9)	49 (7.7)	
Mountain	670 (4.9)	500 (4.3)	154 (3.4)	42 (2.8)	NR^b^	
Pacific	1645 (11.9)	1476 (12.6)	621 (13.8)	213 (14.2)	102 (16.0)	
Year of diagnosis						
2006	234 (1.7)	178 (1.5)	62 (1.4)	29 (1.9)	12 (1.9)	<.001
2007	282 (2.0)	240 (2.0)	94 (2.1)	40 (2.7)	26 (4.1)	
2008	466 (3.4)	385 (3.3)	167 (3.7)	60 (4.0)	17 (2.7)	
2009	477 (3.5)	417 (3.5)	176 (3.9)	40 (2.7)	22 (3.5)	
2010	1294 (9.4)	953 (8.1)	350 (7.8)	115 (7.6)	47 (7.4)	
2011	1455 (10.5)	1197 (10.2)	442 (9.8)	161 (10.7)	71 (11.1)	
2012	1627 (11.8)	1280 (10.9)	442 (9.8)	121 (8.0)	58 (9.1)	
2013	1882 (13.6)	1437 (12.2)	510 (11.4)	192 (12.8)	68 (10.7)	
2014	1839 (13.3)	1578 (13.4)	561 (12.5)	189 (12.6)	79 (12.4)	
2015	2097 (15.2)	1861 (15.8)	770 (17.2)	251 (16.7)	114 (17.9)	
2016	2151 (15.6)	2224 (18.9)	915 (20.4)	306 (20.3)	123 (19.3)	

Abbreviations: IQR, interquartile range; NR, not reported; PSA, prostate-specific antigen; SDT, surgical delay time.

SI conversion factor: To convert PSA to μg/L, multiply by 1.

^a^ Percentages may not always add up to 100% because of rounding.

^b^ Cells have been deleted per National Cancer Database requirements to censor cells containing fewer than 11 observations or other cells that make such cells calculable.

^c^ Educational level was based on number of adults who did not graduate from high school in the patient’s area of residence.

^d^ Income level was based on the median household income in each patient’s area of residence.

### Adverse Pathological Outcomes

**Figure 2.**
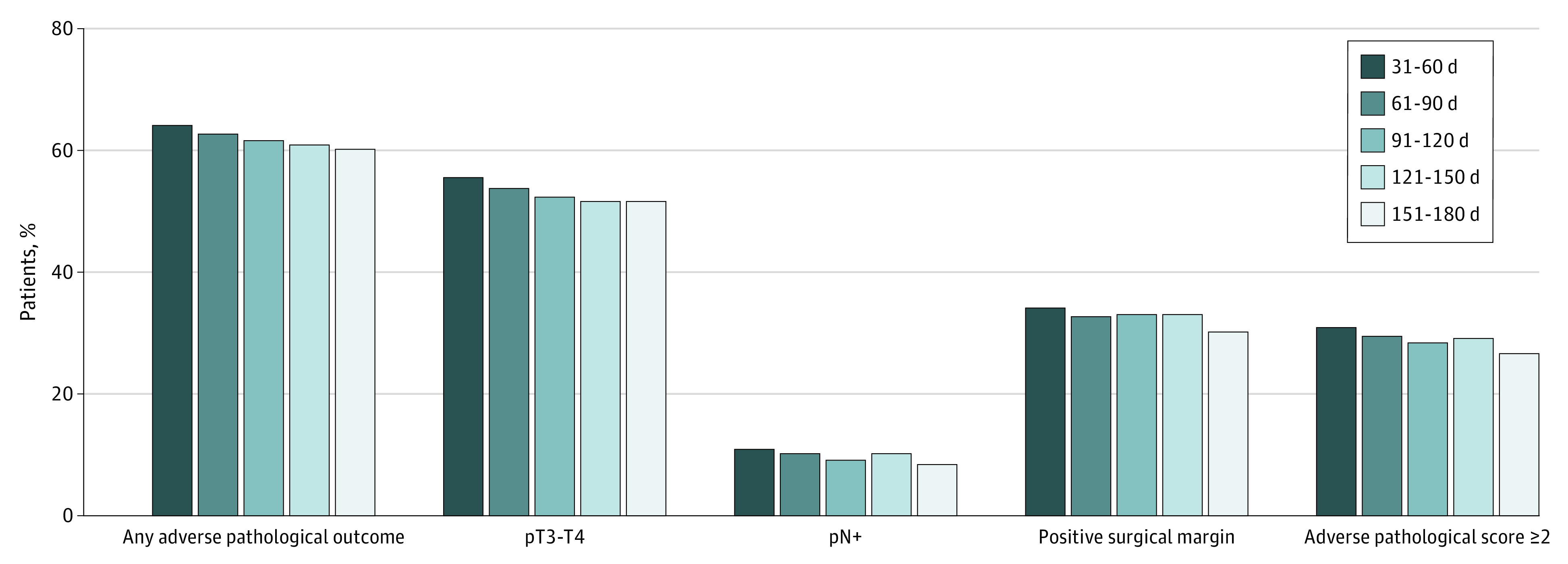
Adverse Pathological Outcomes Stratified by Surgical Delay Time in the Overall High-Risk Cohort

Overall, 20 276 (63.0%) patients had at least 1 adverse pathological outcome, including 10 646 (33.1%) with an APS of 1, 7922 (24.6%) with an APS of 2, and 1708 (5.3%) with an APS of 3. The pT3-T4, pN-positive, and PSM rates were 54.2% (17 453 patients), 10.6% (3401 patients), and 33.4% (10 760 patients), respectively. Adverse pathologic outcomes stratified by SDT are shown in Figure 2.

Multivariable logistic regression results are presented in Table 2. Compared with an SDT of 31 to 60 days, longer SDTs were not associated with higher risks of having any adverse pathological outcomes (odds ratio [OR], 0.95; 95% CI, 0.80-1.12; *P* = .53), pT3-T4 disease (OR, 0.99; 95% CI, 0.83-1.17; *P* = .87), pN-positive disease (OR, 0.79; 95% CI, 0.59-1.06; *P* = .12), PSM (OR, 0.88; 95% CI, 0.74-1.05; *P* = .17), or APS greater than or equal to 2 (OR, 0.90; 95% CI, 0.74-1.05; *P* = .17). It was notable that an SDT of 61 to 90 days was associated with lower odds of having pN-positive disease (odds ratio, 0.85; 95% CI, 0.76-0.96; *P* = .009), which was likely associated with selection bias and statistical variability. Sensitivity analyses also did not show the increased odds of adverse pathologic outcomes with increasing SDT (eTable 1 in the Supplement).

**Table 2.** Multivariable Logistic Regression Results Showing the Associations Between Surgical Delay Time and Adverse Pathological Outcomes in the Overall High-Risk Cohort and Very High-Risk Cohort^a^

Surgical delay time, d	Patients, No.	Any adverse pathological outcome		pT3-T4		pN-positive		PSM		APS ≥2	
		OR (95% CI)	*P* value	OR (95% CI)	*P* value	OR (95% CI)	*P* value	OR (95% CI)	*P* value	OR (95% CI)	*P* value
Overall high-risk cohort (n = 32 184)											
31-60	13 804	1 [Reference]		1 [Reference]		1 [Reference]		1 [Reference]		1 [Reference]	
61-90	11 750	0.98 (0.93-1.04)	.53	0.98 (0.93-1.03)	.42	0.97 (2.89-1.05)	.46	0.98 (0.82-1.03)	.38	0.97 (0.92-1.03)	.36
91-120	4489	0.95 (0.88-1.02)	.14	0.94 (0.87-1.01)	.09	0.85 (0.76-0.96)	.009	0.99 (0.91-1.06)	.69	0.93 (0.86-1.00)	.06
121-150	1504	0.98 (0.87-1.10)	.67	0.98 (0.87-1.09)	.67	1.00 (0.83-1.20)	.97	0.99 (0.88-1.12)	.91	1.01 (0.90-1.15)	.82
151-180	637	0.95 (0.80-1.12)	.53	0.99 (0.83-1.17)	.87	0.79 (0.59-1.06)	.12	0.88 (0.74-1.05)	.17	0.90 (0.74-1.08)	.25
Very-high-risk cohort (n = 2348)											
31-60	1227	1 [Reference]		1 [Reference]		1 [Reference]		1 [Reference]		1 [Reference]	
61-90	791	0.85 (0.68-1.07)	.18	0.91 (0.73-1.12)	.35	0.93 (0.72-1.19)	.55	0.99 (0.82-1.19)	.88	0.98 (0.81-1.18)	.81
91-120	223	0.92 (0.64-1.34)	.67	0.95 (0.68-1.34)	.78	0.76 (0.49-1.16)	.20	0.90 (0.66-1.22)	.49	0.79 (0.58-1.08)	.14
121-150	77	1.96 (0.96-4.01)	.06	1.47 (0.82-2.62)	.20	1.51 (0.84-2.70)	.17	1.31 (0.80-2.14)	.28	1.64 (0.99-2.70)	.05
151-180	30	0.72 (0.31-1.67)	.44	0.71 (0.32-1.56)	.39	1.07 (0.38-2.98)	.90	0.84 (0.38-1.83)	.65	0.75 (0.34-1.69)	.49

Abbreviations: APS, adverse pathological score; OR, odds ratio; PSM, positive surgical margin.

^a^ Adjusted for age, race/ethnicity, Charlson-Deyo comorbidity score, insurance, income level, education level, county type, travel distance, clinical T stage, prostate-specific antigen level, Gleason score, facility type, facility location, and year of diagnosis.

### Survival Outcome

**Figure 3.**
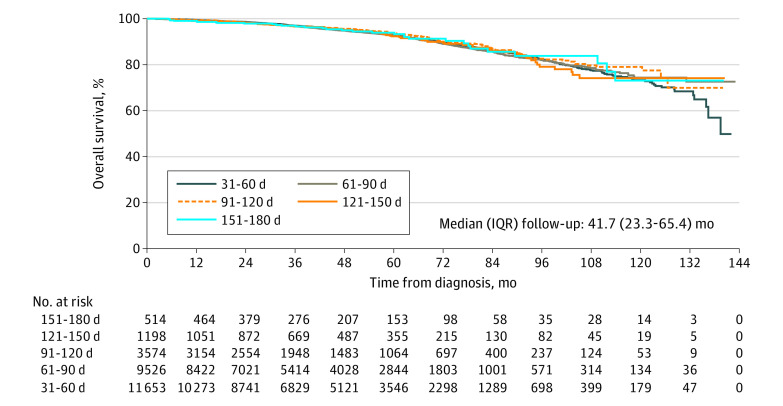
Overall Survival Estimates Stratified by Surgical Delay Time in the Overall High-Risk Cohort Overall survival data were not available for cases diagnosed in 2016. IQR indicates interquartile range.

OS estimates stratified by SDT based on Kaplan-Meier curves in the overall high-risk cohort are shown in Figure 3. The median (interquartile range) follow-up was 41.7 (23.3-65.4) months. A total of 1713 (6.5%) deaths were seen in this cohort, including 767 (6.6%) in the SDT of 31 to 60 days group, 623 (6.5%) in the SDT of 61 to 90 days group, 211 (5.9%) in the SDT of 91 to 120 days group, 79 (6.6%) in the SDT of 121 to 150 days group, and 33 (6.4%) in the SDT of 151 to 180 days group. Multivariable Cox regression results showing the associations between SDT and OS in the overall high-risk cohort and very-high-risk cohort are presented in eTable 2 in the Supplement. Compared with SDT of 31 to 60 days, longer SDTs were not associated with higher risks of death (hazard ratio, 1.12; 95% CI, 0.79-1.59, *P* = .53). Sensitivity analyses also did not show worse OS with increasing SDT (eTable 3 in the Supplement).

## Discussion

In this cohort study using data from a large national database, increased SDT within 180 days of a diagnosis of high-risk prostate cancer was not associated with higher risks of adverse pathological features or lower OS. The findings were consistent for a subgroup of patients with very-high-risk disease (primary Gleason score 5). To the best of our knowledge, this is the largest study specifically evaluating the impact of surgical delay on the pathological outcomes of high-risk prostate cancers in the literature.^4,17,19,20^

Previous studies^4,17,19,21,22,23^ evaluating associations of SDT with postprostatectomy outcomes have 2 common features. First, they included patients with intermediate-risk and low-risk cancer, which might contaminate the findings for high-risk cancers.^4,19^ Risk stratification is crucial for prostate cancer because management and outcomes can vary significantly, especially in the active surveillance era.^9,10,11^ Some of the studies were designed to assess the safety of active surveillance for lower risk cancers.^22,23^ Although some studies did subgroup analyses for high-risk patients, the sample sizes were small, which might contribute to the inconsistent conclusions between studies, with some showing associations between SDT and postprostatectomy outcomes and some not.^17,23,24,25^ Second, previous studies also had to compromise by categorizing SDT into only 2 or 3 groups with large time intervals because of limited sample sizes.^4,17,21,25^ For example, both Gupta et al^17^ and Awasthi et al^21^ compared the outcomes after radical prostatectomy between less than 3 months and 3 to 6 months after diagnosis. The drawback of this type of design is increased bias from categorizing a continuous variable (SDT).

Recently, using the NCDB, Ginsburg et al^20^ reported that delayed radical prostatectomy is not associated with adverse oncological outcomes. They included patients with intermediate-risk and high-risk prostate cancer. In their study, immediate radical prostatectomy was defined as radical prostatectomy within 3 months of diagnosis, whereas delayed radical prostatectomy was analyzed in 3-month intervals up to 12 months. Compared with previous studies, we were able to focus not only on the most aggressive cancers but also have 5 groups of SDT with only a 30-day cutoff. We were able to more finely evaluate any potential differences or associations of the length of time delays from cancer treatment. Categorizing SDT makes the data more interpretable and applicable to the readership. Of note, sensitivity analyses with SDT considered as a continuous variable also did not show worse outcomes with increasing SDT.

Patients face a difficult and complex decision in choosing their treatment, with varying risks for complications, quality of life, and treatment regret. Patient and partner fear and anxiety following a new diagnosis of prostate cancer have been well described in the literature,^26,27^ and are certainly heightened during the ongoing COVID-19 pandemic with the potential delay of treatment. Given the well-established safety of active surveillance for patients with very-low-risk, low-risk, and some intermediate-risk prostate cancers, clinicians can confidently delay definitive surgical treatment in these patients.^28,29^ For newly diagnosed high-risk and very-high-risk prostate cancer, the current evidence is less convincing. Our data support our hypothesis that prolonged time to surgery is unlikely to have adverse effects on oncological outcomes in these men. We believe that this fills a significant gap in the current literature and facilitates a shared decision-making process, especially during the ongoing COVID-19 pandemic.

Although neoadjuvant androgen deprivation therapy (ADT) has a role for patients who plan to undergo definitive radiotherapy for treatment of their localized prostate cancer, there is currently no level 1 evidence to support its use before radical prostatectomy.^30,31^ However, during the waiting time between the diagnosis of high-risk prostate cancer and the point at which the federal ban on elective surgery is lifted, clinicians may consider the use of ADT as a temporizing measure.^2^ In our cohort, we excluded patients who received neoadjuvant ADT yet still saw no compromise in pathological outcomes. Therefore, patients should be counseled that delays in definitive surgical management are feasible without the use of neoadjuvant therapies if there is a COVID-19 resurgence.

The broader implication of our study should not be neglected. For localized high-risk prostate cancer, patients are generally offered multiple options (surgery vs radiation with or without ADT), and a substantial number of patients with prostate cancer seek multiple opinions.^32^ Deciding definitive management for high-risk prostate cancer can be challenging and time-consuming. Our study adds to the literature regarding the safe window of delaying treatment and patients can be assured that they should take a reasonable amount of time before making the final decision. Taking the time to apply thorough patient-centered preference assessments can improve satisfaction with care among patients with localized prostate cancer.^33^ From a health care policy standpoint, our study can provide quality benchmarks for prostate cancer care, especially surgical care. Although this is a study based on US data, the results can also be helpful for other countries with long waiting times for surgery, such as Canada.^25^

### Limitations

This study has some limitations. First, selection bias is unavoidable for retrospective studies and our study is no exception. Also, studies on surgical waiting time are likely to have obvious selection bias because physicians tend to be good at identifying who needs surgery sooner.^15,16^ There was evidence that higher risk patients underwent radical prostatectomy earlier in our study. Although we tried to minimize the bias by excluding patients with SDTs of 30 days or less and adjusting many variables in our study, confounding factors not included in NCDB still exist, and the causes of delay are unknown. Bias could also be introduced by excluding missing data. Second, true long-term cancer-specific outcomes, such as biochemical recurrence, metastasis-free survival, and cancer-specific survival, are not available in the NCDB; therefore, we could only use pathological outcomes as surrogates. Although these pathological features are associated with cancer outcomes, they are not necessarily exclusively related to cancer progression. Both PSM and pN-positive disease can be associated with surgeon experience and other factors (eg, pathologists). Third, follow-up time for OS was short, and this secondary outcome should be interpreted with extra caution. Fourth, only cT1-T2 disease was included and it was unclear what staging method was used (digital rectal examination vs imaging). It is obvious that our results should not be used for decisions regarding locally advanced (cT3-T4) cancer or SDT beyond 6 months. Also, the results may not extend to those who receive radiation therapy. Fifth, the NCDB is a hospital-based database, rather than a population-based database, and patients were treated at Commission on Cancer–accredited facilities, so the results may not be completely representative at the population level.

## Conclusions

In this large, contemporary cohort study of 32 184 patients with clinically high-risk localized prostate cancer who underwent radical prostatectomy within 180 days of diagnosis, increased SDT was not associated with higher risks of adverse pathological features or lower OS. Radical prostatectomy for high-risk prostate cancer could be safely delayed up to 6 months after diagnosis.

## Notes

Supplement. **eTable 1.** Multivariable Logistic Regression Results Showing the Associations Between Surgical Delay Time and Adverse Pathologic Outcomes in the Overall High-Risk Cohort and Very High-Risk Cohort, With Surgical Delay Time Considered as a Continuous Variable **eTable 2.** Multivariable Cox Regression Results Showing the Associations Between Surgical Delay Time and Overall Survival in the Overall High-Risk Cohort and Very High-Risk Cohort **eTable 3.** Multivariable Cox Regression Results Showing the Associations Between Surgical Delay Time and Overall Survival in the Overall High-Risk Cohort and Very High-Risk Cohort, With Surgical Delay Time Considered as a Continuous Variable
