# Supplementary material for: Surgical Delay and Pathological Outcomes for Clinically Localized High-Risk Prostate Cancer
Source: JAMA Netw Open. 2020 Dec 8;3(12):e2028320. doi: 10.1001/jamanetworkopen.2020.28320 (PMC7724561; doi:10.1001/jamanetworkopen.2020.28320)
Supplement: Supplement. — eTable 1. Multivariable Logistic Regression Results Showing the Associations Between Surgical Delay Time and Adverse Pathologic Outcomes in the Overall High-Risk Cohort and Very High-Risk Cohort, With Surgical Delay Time Considered as a Continuous Variable eTable 2. Multivariable Cox Regression Results Showing the Associations Between Surgical Delay Time and Overall Survival in the Overall High-Risk Cohort and Very High-Risk Cohort eTable 3. Multivariable Cox Regression Results Showing the Associations Between Surgical Delay Time and Overall Survival in the Overall High-Risk Cohort and Very High-Risk Cohort, With Surgical Delay Time Considered as a Continuous Variable [file jamanetwopen-e2028320-s001.pdf]

Supplemental Online Content

Xia L, Talwar R, Chelluri RR, Guzzo TJ, Lee DJ. Surgical delay and pathological outcomes for clinically localized high-risk prostate cancer. *JAMA Netw Open*. 2020;3(12):e2028320. doi:10.1001/jamanetworkopen.2020.28320

eTable 1. Multivariable Logistic Regression Results Showing the Associations Between Surgical Delay Time and Adverse Pathologic Outcomes in the Overall High-Risk Cohort and Very High-Risk Cohort, With Surgical Delay Time Considered as a Continuous Variable

eTable 2. Multivariable Cox Regression Results Showing the Associations Between Surgical Delay Time and Overall Survival in the Overall High-Risk Cohort and Very High-Risk Cohort

eTable 3. Multivariable Cox Regression Results Showing the Associations Between Surgical Delay Time and Overall Survival in the Overall High-Risk Cohort and Very High-Risk Cohort, With Surgical Delay Time Considered as a Continuous Variable

This supplemental material has been provided by the authors to give readers additional information about their work.

eTable 1. Multivariable Logistic Regression Results Showing the Associations Between Surgical Delay Time and Adverse Pathologic Outcomes in the Overall High-Risk Cohort and Very High-Risk Cohort, With Surgical Delay Time Considered as a Continuous Variable [a]

**Overall high-risk cohort (n=32,184)**										
	**Any AP**		**pT3-T4**		**pN+**		**PSM**		**APS ≥ 2**	
	OR (95%CI)	P-value	OR (95%CI)	P-value	OR (95%CI)	P-value	OR (95%CI)	P-value	OR (95%CI)	P-value
**Surgical delay time, (per 30-day)**	0.98 (0.95-1.00)	0.109	0.98 (0.96-1.01)	0.117	0.94 (0.90-0.98)	0.004	0.98 (0.95-1.00)	0.056	0.96 (0.94-0.99)	0.009
**Very high-risk cohort (n=2,348)**										
	**Any AP**		**pT3-T4**		**pN+**		**PSM**		**APS ≥ 2**	
	OR (95%CI)	P-value	OR (95%CI)	P-value	OR (95%CI)	P-value	OR (95%CI)	P-value	OR (95%CI)	P-value
**Surgical delay time, (per 30-day)**	1.00 (0.89-1.13)	0.975	1.02 (0.91-1.14)	0.779	0.99 (0.87-1.13)	0.895	0.98 (0.89-1.09)	0.770	0.99 (0.89-1.10)	0.845

Abbreviations: AP, adverse pathology; APS, adverse pathologic score; CI, confidence interval; OR, odds ratio; PSM, positive surgical margin.

[a] Adjusted for age, race/ethnicity, Charlson-Deyo (comorbidity) score, insurance, income level, education level, county type, travel distance, clinical T stage, PSA level, Gleason score, facility type, facility location, and year of diagnosis.

© 2020 Xia L et al. *JAMA Network Open.*

eTable 2. Multivariable Cox Regression Results Showing the Associations Between Surgical Delay Time and Overall Survival in the Overall High-Risk Cohort and Very High-Risk Cohort[a]

**Surgical delay time, days**	**Overall high-risk cohort (n=26,465)**		**Very high-risk cohort (n= 1,897)**	
	HR (95%CI)	P-value	OR (95%CI)	P-value
**31-60**	Reference		Reference	
**61-90**	1.07 (0.96-1.19)	0.205	1.27 (0.96-1.68)	0.097
**91-120**	0.99 (0.85-1.15)	0.870	0.98 (0.61-1.58)	0.935
**121-150**	1.13 (0.89-1.43)	0.325	0.66 (0.24-1.81)	0.416
**151-180**	1.12 (0.79-1.59)	0.531	1.08 (0.33-3.51)	0.899

Abbreviations: CI, confidence interval; HR, hazard ratio.

[a] Adjusted for age, race/ethnicity, Charlson-Deyo (comorbidity) score, insurance, income level, education level, county type, travel distance, clinical T stage, PSA level, Gleason score, facility type, facility location, and year of diagnosis. Overall survival data not available for cases diagnosed in 2016.

© 2020 Xia L et al. *JAMA Network Open.*

eTable 3. Multivariable Cox Regression Results Showing the Associations Between Surgical Delay Time and Overall Survival in the Overall High-Risk Cohort and Very High-Risk Cohort, With Surgical Delay Time Considered as a Continuous Variable[a]

	**Overall high-risk cohort (n=26,465)**		**Very high-risk cohort (n= 1,897)**	
	HR (95%CI)	P-value	OR (95%CI)	P-value
**Surgical delay time, (per 30-day)**	1.02 (0.97-1.08)	0.393	1.01 (0.86-1.18)	0.946

Abbreviations: CI, confidence interval; HR, hazard ratio.

[a] Adjusted for age, race/ethnicity, Charlson-Deyo (comorbidity) score, insurance, income level, education level, county type, travel distance, clinical T stage, PSA level, Gleason score, facility type, facility location, and year of diagnosis. Overall survival data not available for cases diagnosed in 2016.

© 2020 Xia L et al. *JAMA Network Open.*
